# Leveraging LoRaWAN Technology for Precision Agriculture in Greenhouses

**DOI:** 10.3390/s20071827

**Published:** 2020-03-25

**Authors:** Ritesh Kumar Singh, Michiel Aernouts, Mats De Meyer, Maarten Weyn, Rafael Berkvens

**Affiliations:** IDLab—Faculty of Applied Engineering, University of Antwerp—imec, Sint-Pietersvliet 7, 2000 Antwerp, Belgium; michiel.aernouts@uantwerpen.be (M.A.); mats.demeyer@uantwerpen.be (M.D.M.); maarten.weyn@uantwerpen.be (M.W.); rafael.berkvens@uantwerpen.be (R.B.)

**Keywords:** precision agriculture, WSN, greenhouse, LoRaWAN

## Abstract

The technology development in wireless sensor network (WSN) offers a sustainable solution towards precision agriculture (PA) in greenhouses. It helps to effectively use the agricultural resources and management tools and monitors different parameters to attain better quality yield and production. WSN makes use of Low-Power Wide-Area Networks (LPWANs), a wireless technology to transmit data over long distances with minimal power consumption. LoRaWAN is one of the most successful LPWAN technologies despite its low data rate and because of its low deployment and management costs. Greenhouses are susceptible to different types of interference and diversification, demanding an improved WSN design scheme. In this paper, we contemplate the viable challenges for PA in greenhouses and propose the successive steps essential for effectual WSN deployment and facilitation. We performed a real-time, end-to-end deployment of a LoRaWAN-based sensor network in a greenhouse of the ’Proefcentrum Hoogstraten’ research center in Belgium. We have designed a dashboard for better visualization and analysis of the data, analyzed the power consumption for the LoRaWAN communication, and tried three different enclosure types (commercial, simple box and airflow box, respectively). We validated the implications of real-word challenges on the end-to-end deployment and air circulation for the correct sensor readings. We found that temperature and humidity have a larger impact on the sensor readings inside the greenhouse than we initially thought, which we successfully solved through the airflow box design.

## 1. Introduction

Wireless sensor network (WSN) technology has rapidly evolved over the years enabling a spectrum of applications such as military, industry, agriculture and healthcare [1]. WSNs provide favorable facilities for agriculture applications through cost-effective process leading to increase in crop yield. It facilitates farmers to minimize the wastage of pesticides, effective control of pests and disease as well as supplying adequate amount of nutrients for precision agriculture (PA). This, in turn, improves agricultural production, quality and most crucial identifies the variation in microclimate and maps with the management activity [2]. Vast areas of agricultural land can be monitored using sensor nodes which can forward data through wireless communication to a receiving gateway. WSNs can be used in sub-divisions of agricultural applications such as forecasting the health of the crop, the guarantee of adequate amount of nutrients, disease detection, irrigation planning, and climate monitoring [3]. The diverse sensor nodes and their respective communication links provide precise information of the field by monitoring a wide range of environmental parameters required for PA. Therefore, there has been continuous research contributions towards enriching WSNs capabilities like communication, sensing and processing power of sensors nodes [4].

**Figure 1:**
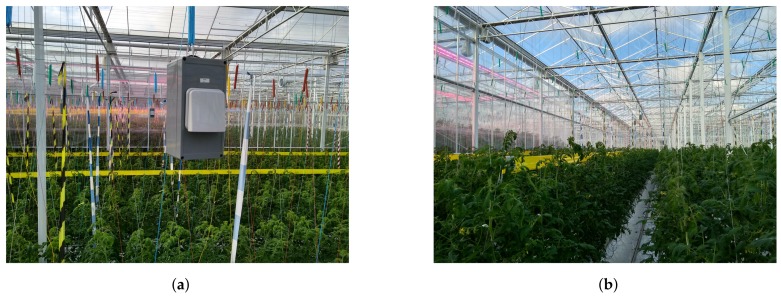
Layout of the greenhouse (PCH research center) where we conducted our measurements with our deployed sensor box. (**a**) Rectangular box as mounted in the greenhouse; (**b**) rows of tomato plants.

Several research contributions have been done regarding PA, mainly in the area of data collection strategies, data analysis, diagnosis of several parameters along with forecasting disease, field operation and evaluation of precision agriculture techniques [3,5]. The precision agriculture employs a cost-effective management strategy using information technology in two ways: first by identification of spatial variation and addressing appropriate statutory activities. Second, by controlling the usage of weeds, pesticides, and diseases to proliferate crop yield [6]. Greenhouse agriculture and cultivation demand an efficient scheme for controlling microclimate conditions such as temperature, humidity, and gas concentration to maintain the ambient setup for crop cultivation. Greenhouse embellish effectiveness against harsh climates and impediment faced with outdoor cultivation [7] by providing mechanical shields which help in maintaining crop-specific reasonable environmental conditions. The operations in greenhouses depend on the technologies used for controlling climatic parameters, the capacity of greenhouse (shape and dimensions), covering material and its orientation [8]. Ideally, the design structure should keep a uniform climate profile across the greenhouse. However, greenhouse faces a handful of challenges, majorly due to complex structural design scheme [9] as shown in Figure 1. Also, there are other challenges like the design adaptation as per the crop change, impact of metallic structures in greenhouse and the technology used in the greenhouses for PA as listed in Table 1. These challenges have a substantial footprint over the growth of a plant, which necessitates a precise and adaptive monitoring solution. WSNs provide an effective solution for PA but on another side, it requires an efficient strategy in different fundamental aspects like connectivity, type of sensors, power source, and network optimization [4].

WSNs provide a cost-effective approach for PA in greenhouses [10]. Different applications such as climate monitoring, irrigation planning, feeding recommended nutrition and forecasting crop health require a diversified control system with a wide range of sensing capabilities. Table 2, exhibits the different types of sensors and control systems used inside the greenhouse for various use cases like tracking, monitoring and controlling environmental conditions. However, there are some challenges in WSN deployment which has curtailed the real benefits for PA; such as an optimal deployment scheme, maintain coverage and connectivity for required communication range, scalability, and energy-efficient network for long battery life [11].

The sensor nodes are mostly battery powered. Thus, the right selection of low-power sensors and communication network is imperative for PA. Most of the solutions used Zigbee [12] to transmit data, but a trade-off must be made between scalability and reliability. Also, a major disadvantage is the relatively short transmission range of 100 m, which is not well suited for large greenhouses [12]. LPWANs are best suited wireless communication for PA in greenhouses due to their low power consumption and long communication distance [13]. One of the promising protocols in this scope is LoRaWAN [14]. This protocol uses Long-Range (LoRa) modulation [15] in its physical layer and features a low data rate with low complexity and long coverage. Recently, a case study has been done for PA using a LoRaWAN network for viticulture and greenhouses [16,17], and a more specific micro use case like smart irrigation [18]. It specifically highlights the importance of accurate readings from sensors in a greenhouse along with a requirement for better data visualization and analysis.

This paper describes the development and deployment of the LoRaWAN network to monitor environmental conditions in a greenhouse. This system is deployed at the research center Hoogstraten, which is in the north of the province of Antwerp, Belgium. It has multiple greenhouses; our research was conducted in a greenhouse with tomato crops. Deployed sensor network monitors environmental parameters such as temperature, humidity, carbon dioxide (CO_2_), electrical conductivity (EC) and illuminance. These sensors are battery operated and use private LoRaWAN network to forward the data to a gateway that is installed at the research center. This data is used by the biologists in the project (KU Leuven and University of Antwerp, Belgium) to develop crop models and perform disease prediction. Also, this data is helpful for the technicians in the greenhouse to monitor the growth of tomato. The sensor data is visualized on our customized ThingsBoard dashboard. The deployment was done in three phases to understand the impact of different sensor box designs in reading sensor values. These boxes are necessary to protect the sensors against adversary weather conditions and from regular spray inside the greenhouse [19]. Initially, we deployed off the shelf sensors and mounted inside the normal strawberry boxes which got affected due to daily spray. The second round, we deployed sensors inside in house designed box. This was again not giving the correct sensor values due to temperature difference inside the enclosed box, with limited airflow. Lastly, we built our customized airflow boxes which had proper air circulation to achieve the correct measurement of sensor values.

The main contribution of this paper is three-fold. First, this paper summarizes the prospects and challenges for precision agriculture in greenhouses, along with salient considerations (learning from our experiment) for WSN deployment in the greenhouse. Also, we propose the successive steps to be followed for optimal WSN deployment in the greenhouse. Second, this paper illustrates the importance of the design scheme of a sensor box to get the accurate sensor readings. This is achieved by using the airflow box as sensor enclosures in the greenhouse. Third, we show our end-to-end LoRaWAN-based WSN system for greenhouse monitoring, along with power analysis of LoRaWAN communication and our data visualization dashboard using ThingsBoard.

The rest of the paper is organized in the following way. Section 2, describes the current state of the art for precision agriculture in greenhouses. The evolution of energy-efficient WSNs and its integration with LPWAN in the context of PA. Section 3, explains the system architecture along with material, methods and sensor deployment used for the current setup in the greenhouse. Section 4 presents the proposed dashboard provisioned with data visualization, sensor location, and other feature sets. In Section 5, we discuss the current results over the data variation in the greenhouse. Finally, we conclude the paper in Section 6, highlighting potential future work.

## 2. Related Work

Greenhouse technology is widely accepted as a crucial part of agricultural engineering. Early in 1994 Blackmore et al. [20], mentioned the importance of monitoring soil and environment for quality yield. Thereafter, there has been continuous research on the integration of WSNs in greenhouses. Initially, farmers were reluctant to use WSNs because of the cost but with the progressive technology development, the overall cost has gone remarkable down [21]. WSNs provide monitoring for precision agriculture in the greenhouse using multi-parameter monitoring [22] and using low-power and reliable wireless communication technology. The communication technology and the right selection of application-specific sensor nodes are an integral part of WSN for PA in greenhouses. There have been several contributions [23,24] to lower the management cost with the help of agricultural policy and innovative farming methods. Also, for better analysis the sensor data are integrated with the video information for precision effective farming [25]. However, it is equally crucial to access the risks associated with the new agricultural practices before its adoption on the field [26].

WSNs are mostly battery constrained, the average battery life of a wireless node in precision agriculture use case lasts for only a few months [3]. However, the desired power consumption should stretch the battery life for at-least more than a year. This requires a taxonomy of energy-efficient and energy harvesting techniques as mentioned by author Jawad et al. in their review article on energy-efficient WSNs for PA [10]. There have been remarkable research contributions in power reduction techniques in WSNs by exploiting radio schemes, routing and optimizing link connectivity for long term monitoring [27]. In greenhouses, hygiene is maintained with high priority to prevent the spread of pathogen agent that can cause disease to crop. Therefore, the demand for self-maintenance and long battery life plays a vital role in greenhouses to avoid frequent hardware visits. Ideally, even if the node fails or the battery dies, the network coverage should be maintained. For realizing this scenario, research contribution has been done in different schemes like clustering, role-based approach, etc. [28]. Another prototype has been introduced for maintaining equal use of energy among different nodes by combining energy harvesting with dynamic job switching of sensors based on left energy [29]. With the requirement of less space and more production [30], the adaptation of LPWANS for transmitting data over long distance with low cost is becoming considerate [31]. In parallel, new energy harvesting methods [32] are to be explored as existing solutions like solar panels, etc. [33]. are difficult to be used in greenhouses due to complex covered hardware topology [34].

LoRaWAN is an open standard which uses LoRa modulation to enable long-range and low-cost solution with optimal power consumption. There has been a growing interest of using LoRaWAN for precision agriculture, such as the design of the greenhouse irrigation system (master/slave) [35], case study on open agricultural monitoring in Kenya [36] and also precision farming in viticulture [16]. These use cases require further end-to-end scalable solutions and a remote monitoring dashboard for data visualization.

The complications and prospects for the role of sensor boxes are given in [17]. It states the contradiction of reading values as the temperature is elevated inside the box due to minimal or no air circulation, which affects the humidity reading as well. Also, it states the prospect solution for this problem to be an air-ventilated box, where sensors are either placed outside the box or near the fan for air circulation. Instead of tight packaging, it is recommended by the authors to place the sensors in different parts of the box [37] like humidity sensor near the fan, temperature sensor at the border or out of the box and sound sensor away from the fan. By using any casing, the sensors can be protected from weather conditions [19] but may result in the error in sensor readings.

Computing techniques like machine learning (ML), helps in investigating and analyzing the available data from various fields and integrating them with the process of crop improvement. ML provides various analytical models and methods to analyze the crop disease, yield prediction and so on. The authors Susanto B et al. [38] used the vision sensing approach to estimate nutrient contents in the wheat leaves. Nutrient estimation is necessary to avoid over fertilizing to the crops, which in turn harms the crop as well as environment. Because of the change in intensity of light, it becomes very challenging to estimate nutrition with inconsistent image. To tackle this problem, Susanto B et al. [39], developed combination of neural networks by using color constancy method to normalize different color of images. Neural network-based prediction of soil water is used in the paper [40], for the management of water valves to achieve optimized irrigation. PA generates structured and unstructured datasets, which needs the extraction of knowledgeable information. Paper [41], uses the big data approach to get insights and explore the potential sources of big data in PA. These big data analytics helps in figuring out the trend and pattern which helps in decision making, future farming etc. Also, paper [42], opens the dataset to the community to stimulate research in this area. A review paper on research progress of ML approaches [43], demonstrates the rapid advances in ML techniques for better crop yield using cost-effective decision making. As future work, we will integrate the ML and MAC protocol [44] to secure data transmission and minimize the frequency of messages from sensor node to the gateway in order to achieve energy-efficient network.

Article on IoT-based smart agriculture [45] by the author Muhammad Ayaz et al. identifies the current and future trends of IoT in agriculture. It states that LPWAN technology can be a game changer for smart agriculture and stands as better solution for connectivity because of long-range and affordable price for the farmers. In this paper, we use the LoRaWAN as connectivity enabler for precision agriculture and check the impact of using different box schemes by deployment in phases. Lastly, we realized the final end-to-end LoRaWAN-based deployment by using the air circulated box for the sensors on field. This will be helpful for both researchers and growers in future deployments for precision agriculture.

## 3. Materials, Methods and Field Tests in a Greenhouse

**Figure 2:**
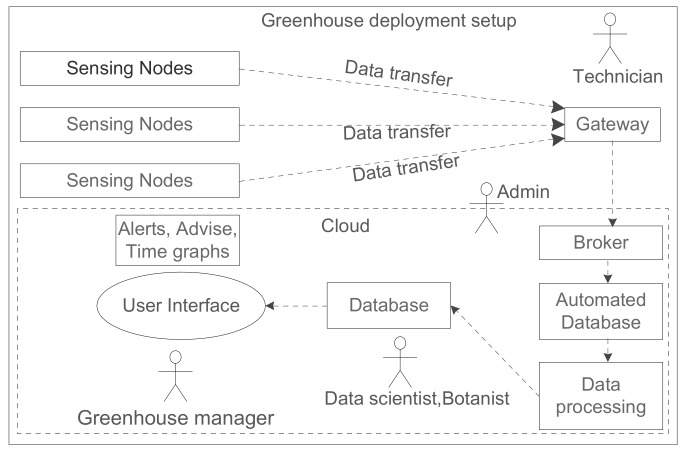
Greenhouse deployment setup.

The experiments were conducted in a research center Hoogstraten (PCH) situated near the province of Antwerp, Belgium [46]. It is one of the most innovative research centers in horticultural, managing 166 trials in the greenhouse over the span of one year [47]. PCH has open-air and greenhouse-based cropping systems mainly for strawberries, bell peppers, and tomatoes. Our experiments were conducted over tomato crop which combines practical research of WSNs deployment, with directly applicable results on the crops. The deployment system architecture is shown in Figure 2. PCH has divided the area into several compartments, with an individual size range of 500 $m^{2}$ to 1000 $m^{2}$ and a height of 8 m.

The first research step was a series of qualitative visits to understand the topology of the greenhouse, crop cycle, growth of the plants, relevant climate parameters to be monitored as part of requirement and possible interference for link connectivity. We defined different phases for the study and monitoring of the crop. This was done with an objective to analyze the different challenges at each stage of deployment such as the variation of sensor values at a different gradient, optimal network coverage, and wireless connectivity that might be relevant for final WSN deployment. The following part of this section reveals the sequential steps that we followed and equally propose this as the potential roadmap for the WSN deployment in the greenhouses.

### 3.1. Steps for WSNs Deployment in a Greenhouse

**Figure 3:**
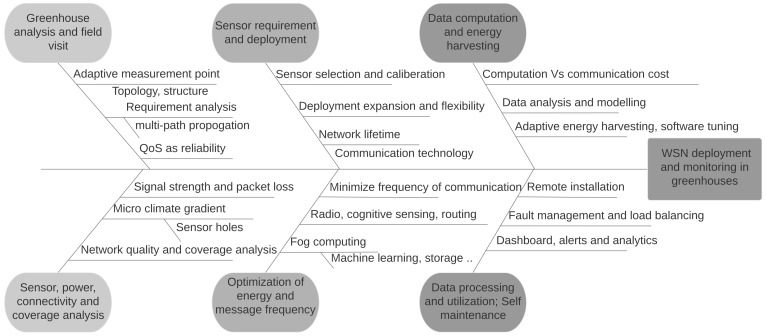
Sequential steps for WSN deployment and monitoring in a greenhouse.

The sequential steps used in this WSN setup for both deployment and research purpose are shown in Figure 3. Based on the challenges in the greenhouse, discussed earlier in Table 1, we propose the ordered steps and the potential research accomplishment in each step for monitoring in greenhouse. The prerequisite for greenhouse monitoring is, to do a field visit and retrofit the requirements of the sensors and their wireless connectivity. The initial study involves analysis of greenhouse complex topology, crop cycle period and plant growth. It gives an idea of what all crop-specific parameters needs to be monitored. There needs to be a trade-off between the frequency of data transmission and the expected battery lifetime of the sensor. In parallel, it is vital to pick the right wireless communication technology concerning distance between sensor node and gateway, power source and number of message transmission. Once the fundamental WSN setup is established, energy optimization can be done in different ways. Most greenhouses follow strict hygiene checks so instead of frequent intrusion for battery replacement or maintenance, it is better to opt for energy harvesting, self-adaptation, and self-maintenance techniques. We will include aforementioned concepts in the solution as part of our future work. There are considerable checkpoints before deployment such as: (1) The installation location of the sensor. (2) Types of sensors that are required to monitor a specific crop. (3) We needed a wireless technology for reliable communication, for which we selected LoRaWAN. (4) Access to the sensor readings using a mobile application or dashboard. (5) Provision for an end-to-end data flows from sensors along with the sensor box to get the accurate readings from sensors.

### 3.2. Communication Technology

Given the large area of the greenhouses in PCH ranging from 500 $m^{2}$ to 1000 $m^{2}$, we wanted a mutually reinforcing LPWAN technology to balance out distance coverage with optimized low power usage. LoRaWAN [14] is a proprietary long-range wireless technology using Chirp Spread Spectrum (CSS) modulation technique named LoRa. In Europe, LoRa operates in the license-free 868 MHz band. Symbols are encoded in the form of chirps over a wider channel bandwidth. This technique curtails the inherit challenge of greenhouse that is reducing interference, multipath and fading effect. The spreading factor (SF) ranges from 7 to 12, providing a balance between data rate and range. Higher SF achieves longer distance with the data rate ranging from 0.3 kbps to 27 kbps. We have multiple channel support in LoRaWAN with a payload of a maximum size between 51 B and 222 B, depending on the SF. The duty cycle is not a primary concern while deploying LoRaWAN-based application in greenhouse [48]. The LoRaWAN module chipset, microcontroller, and LoRa-based configuration used for the deployment is given in Table 3.

### 3.3. Optimal Design for Sensor Box

**Figure 4:**
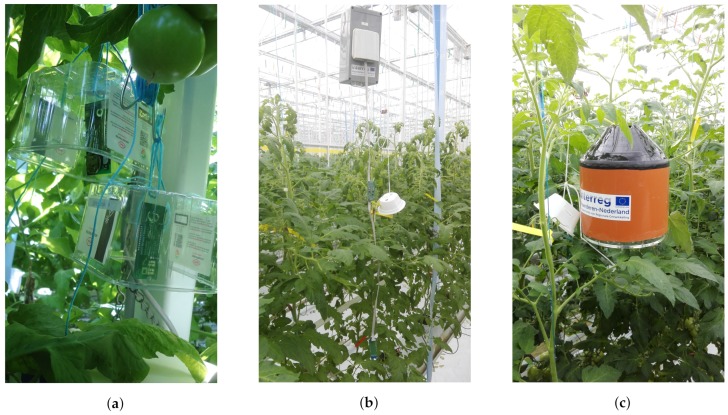
Different types of boxes used for deployment: (**a**) Sensirion sensors protected by strawberry box; (**b**) Rectangular box casing; and (**c**) airflow box casing.

To understand the impact and utility of the sensor box, deployment of the sensors in the greenhouse were performed in three phases. First approach known as Commercial (Sensirion sensors) approach, we deployed a few Sensirion SHT31 development kits at different locations inside the greenhouse. To protect the sensors from regular spray and water, we kept them inside a temporary open box. We have taken the strawberry boxes to mount the sensors as they are as shown in Figure 4a. These boxes have a large open face to expose sensors. The SHT31 kit has humidity and temperature sensors and shows the values on the inbuilt display. Also, it can send the data wirelessly to a smartphone using Bluetooth Low Energy (BLE). The mobile Android application is available open source to visualize the data. We customized this application to download the readings in a file for better analysis. The data logging capabilities helped us to visualize the live data, but the coin cell batteries had a limited lifetime of one week. We learned two important lessons from this initial setup. First, the sampling and sensitivity test in the greenhouse and second, the impact of regular spray and pesticides over the installed sensors. With the help of this initial setup, we got the sampling locations based on the reading gradient in the greenhouse.

**Figure 5:**
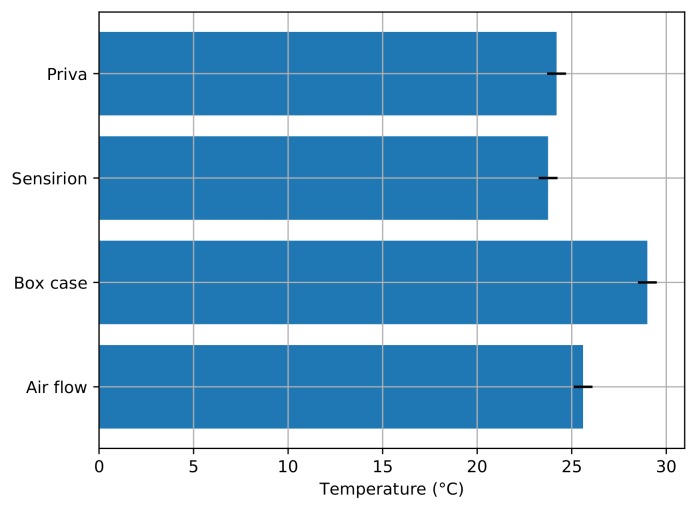
Temperature readings from different boxes.

For the next round of installation, known as box approach, we made an enclosed rectangular box to protect the sensors from external spray. The sensors inside the box were integrated with LoRa module for sending the data to the back end. This box was mounted on the rails above the tomato plants, so that it can be moved freely with the plants’ growth. The provision for air circulation inside the box was done with the opening on the front side as demonstrated by Figure 4b. Post deployment, we again wanted to check the precision of this box in capturing the environmental data. We used thermocouple strip [49] and readings from Priva [50] sensors to compare the temperature of outside and inside the box. We found that the box design still needed some more optimization, as the temperature reported by the Priva sensors was comparatively less from the one inside the box as shown in Figure 5. The temperature reading inside the box reported higher temperature, which was majorly due to the fact of limited air circulation along with two major factors (1) sunlight heating up the box and (2) the heat produced by the sensor board itself. With these results, we wanted to come up with better box design with proper air circulation.

**Figure 6:**
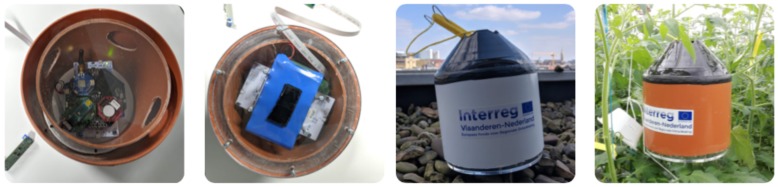
The airflow box.

The final and third approach is known as airflow box approach. Here, the deployment of WSN in the PCH greenhouse was done using the airflow boxes which is shown in Figure 4c. These boxes are circular in shape with an opening on both top and bottom for proper airflow. The Figure 6, shows the steps from building the airflow boxes, till the final deployment in the greenhouse. It has two shells for insulation from outside sun light and opening at the top and bottom for maintaining the air circulation. The respective size for the pipe used for building these boxes were 200 × 3.9 mm and 160 × 3.2 mm. There are two acrylic laser cut plates for covering top and bottom, along with to hold the cross-section of the pipes. The first part of Figure 6, shows the structure of the box from the top view, followed by the placement of sensors and batteries. Thereafter, the box in its deployment stage at the greenhouse.

### 3.4. Architecture Schema of WSN Communication in Greenhouse

**Figure 7:**
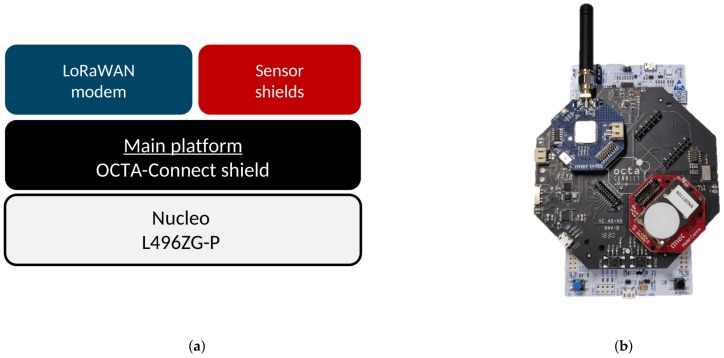
Deployment setup (**a**) Hardware setup overview. (**b**) Example configuration with CO_2_ sensor shield.

In Figure 2, we illustrate the high-level architecture and deployment setup in the greenhouse. It comprises of the on-field deployment of the sensors and gateway and processing of the data at the back end. The hardware setup used for the deployment are shown in Figure 7. This hardware setup is mounted inside the airflow boxes and are deployed in the PCH greenhouse. We used the imec’s OCTA-Connect board stacked on top of Nucleo board. The OCTA-Connect board provides the provision to stack further the wireless connectivity and sensor module. We have connected the LoRa shield and sensors as depicted in Figure 7. This hardware takes the power source from the battery attached inside the airflow box. The LoRaWAN Kerlink gateway [51] is installed inside the greenhouse with an ethernet and power connection. The technician of the greenhouse can install the sensors and gateway as there is no manual configuration required, but just switching on the OCTA-Connect board. While turned on, all the deployed devices automatically start sending data along with battery status. On the back end, the raw sensor data is parsed, stored in MongoDB database following which it is forwarded to a custom ThingsBoard dashboard.

**Figure 8:**
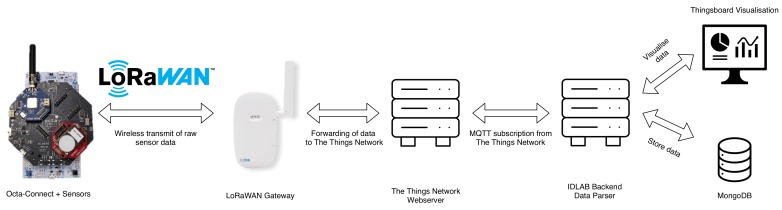
End-to-end data flow from sensors to the dashboard.

The data sequence from reading the sensor values until its visualization is shown in the Figure 8. The OCTA-Connect platform board used in this experiment has several onboard sensors, as well as the ability to stack a variety of shields onto it. The system architecture for the deployment is shown in Figure 2, and overview of the available sensors, both embedded on the OCTA-Connect board and as stackable shields are shown in Table 4. Every setup has a LoRaWAN communication shield attached, of which the configuration is shown in Table 3. The STM32L496ZG Nucleo 144 board is used to program and debug the OCTA-Connect board. The readings of the sensors are taken every five minutes and then forwarded to a Kerlink LoRa gateway. This gateway has a power and ethernet connection, being placed inside the technician office next to greenhouse chambers. The data from the gateway is forwarded to the Things network. At the back end (local database), we subscribe to the data using the MQTT protocol. This data is then used in two ways; to store in the MongoDB and to show the data on the customized ThingsBoard-based dashboard.

## 4. Customized Dashboard and Data Analysis

**Figure 9:**
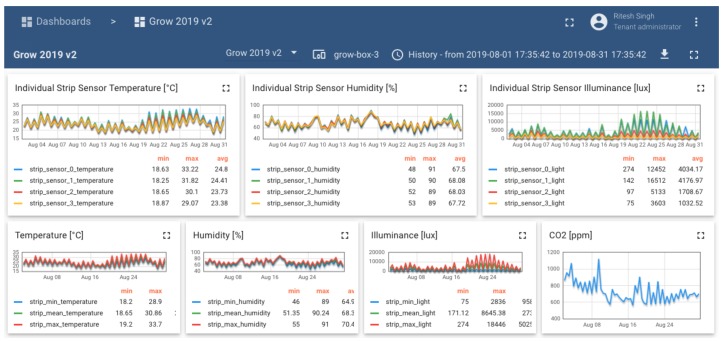
Dashboard showing sensor values for tomato crop (one-month data with message interval of 5 min).

**Figure 10:**
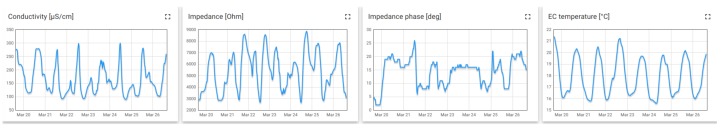
EC reading values.

For the data analysis and visualization, we have used the ThingsBoard customized dashboard as shown in Figure 9 and Figure 10. It allows the stakeholders like people from botany, data researchers and analysts, also growers to perform data mining for their respective use cases. It gives the real-time data for crop health and disease prediction. Also, there is a provision to fetch the box specific historical data over a time period. From security perspective, dashboard allows the admin to define the role of different users. There are two levels of security, the first level is through credential-based authentication and the next level is access-based, i.e., each user can read and write specific sources of data depending on the assigned rights. Post login, a user can select the sensor from the available list of deployed sensors and the option of real-time data or historical data. Also, it provides the provision for visualizing the data for a particular time interval as the mean value. Post selection of the sensor box, the dashboard exhibits the data of all the sensors attached to the respective sensor box. Each sensor box deployed in the greenhouse has numbering which is mapped with its data representation on the dashboard. In the Figure 9, we have selected the Grow box number 3 (airflow box number) with the time interval for full month of August. Therefore, it shows the result as, all the sensor values of the attached sensors with the airflow box number 3 for the given period. For the precision agriculture in the greenhouse, we provide the data seekers with two possibilities to fetch the data. The best case by pulling the data from the dashboard and worst case (scenario where dashboard stops working), we provide a python script to export the sensor data directly from our MongoDB database in a required format.

**Figure 11:**
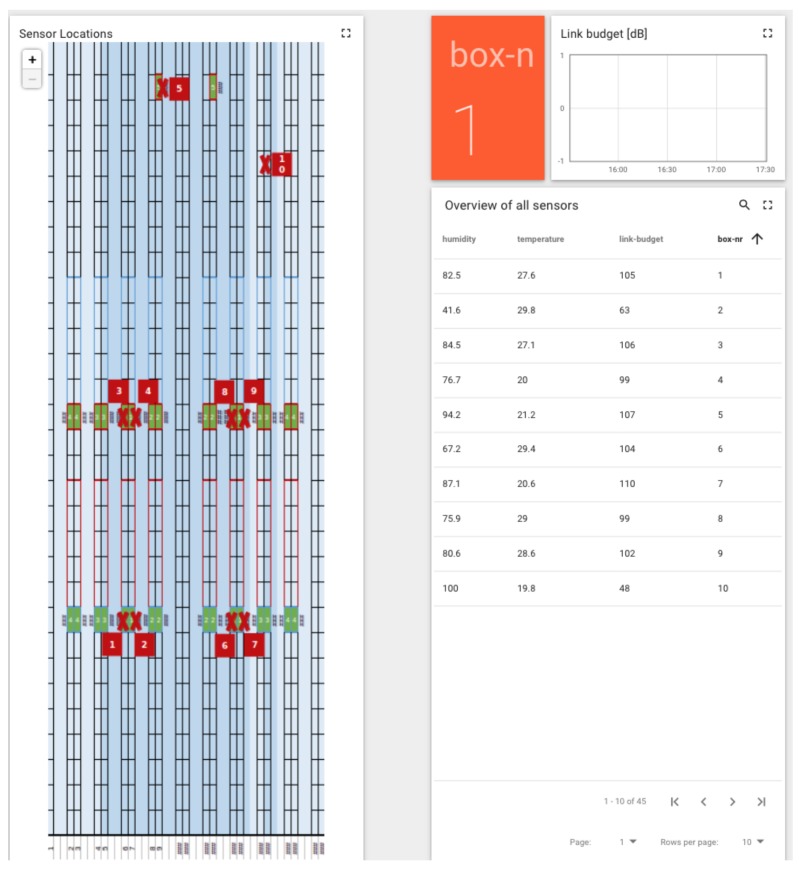
Overview of sensor box and its location in the greenhouse.

Figure 11, is another perspective of the dashboard to visualize the location for the deployed sensor boxes. This helps to see the list of available sensors boxes in the greenhouse along with their link budget, average temperature, humidity and other sensor readings for a given sensor box. The real-time data for one or more sensor boxes can also be demonstrated through the dashboard, it will show the sensor readings for those attached to the respective sensor box. In greenhouses, light plays an important role in plant growth. Therefore, for this scenario, the sensor box reports the data for RGB value, light intensity, temperature and humidity and other readings important for the plant growth.

Figure 10, shows the reading of a water sensor attached to one of the sensor boxes in the greenhouse. Also, the variation of the microclimate readings over a period of one month. This shows the sensor value gradient over the greenhouse. The peak values are mostly observed when the windows of the greenhouse are opened which effects the overall environmental conditions in the greenhouse. For instance, the CO_2_ levels are increased by pumping CO_2_ into the greenhouse and regulate these CO_2_ levels back to normal by opening the windows, and letting cleaner air in from the outside.

## 5. Discussion

**Figure 12:**
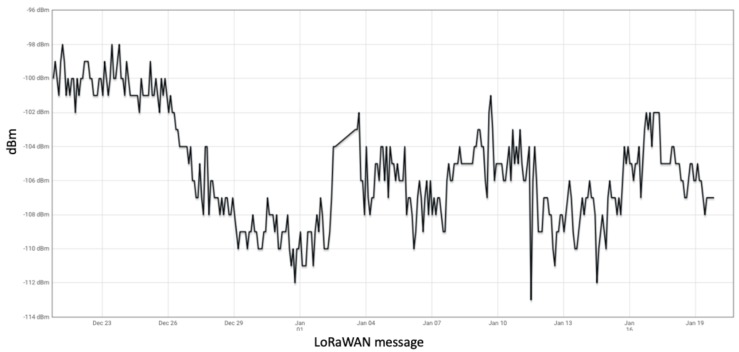
Messages along with signal strength (RSSI) inside greenhouse.

Greenhouse has a complex topology as shown in the Figure 1. Most of the common inherent challenges in the greenhouses are captured in Table 1 along with their respective impact. We proposed the sequential steps to be followed, for the deployment of WSNs in the greenhouse and the benefits it brings at each stage. Deployment of different types of sensor boxes helped to study the impact of the box enclosure on the sensor readings. This is very important and critical consideration while any WSN deployment for the precision agriculture. The evolution of sensor boxes from simple fruit boxes to the new airflow boxes is shown in Figure 4. The airflow boxes help in airflow circulation which concisely contributes to removing the error element in sensor readings. A LoRaWAN setup is deployed in a greenhouse which is shown in Figure 2, along with different components used at different parts of the deployment. This setup helps in monitoring tomato crop in PCH and build the WSN platform based on LoRaWAN for data collection and analysis. The dashboard assists different stakeholders to do the analysis and take necessary action for the growth of plant. Through the data generated from the sensors (interval of 5 min) as in Figure 9 and in Table 5, we see that there is a huge variation of different climatic conditions such as temperature, humidity, CO_2_ and likewise throughout the greenhouse over a period of one month. Therefore, regular monitoring is crucial for the proper growth of the plant. Also, we see the frequent change in the received signal strength indicator (RSSI) for each message in Figure 12, in-spite of the static location of the sensor boxes. It shows the implication of plant growth and other movement inside the greenhouse over signal strength.

### 5.1. Application in the Context of the Rural Farmers

The deployed LoRaWAN-based solution can improve the agriculture by monitoring the field. It can save the efforts and time of the farmers and reduce the excessive usage of resources. This solution is best suited for the rural farmers because of its low cost and ease of deployment. For the deployment of this setup, we first check the LoRaWAN coverage and accordingly decide the requirement of gateway. Both the gateway and sensor devices are made to be plug and play and auto-configurable to the back-end system. We have tested the current system for three tomato growth cycles, and it stood to be stable. We have been sending sensor readings every five minutes to the gateway and plan to minimize the number of transmissions using ML. We plan to extend this setup to other greenhouses and enclosed chambers for monitoring crops like chicory and strawberries.

### 5.2. Power Consumption Analysis

**Figure 13:**
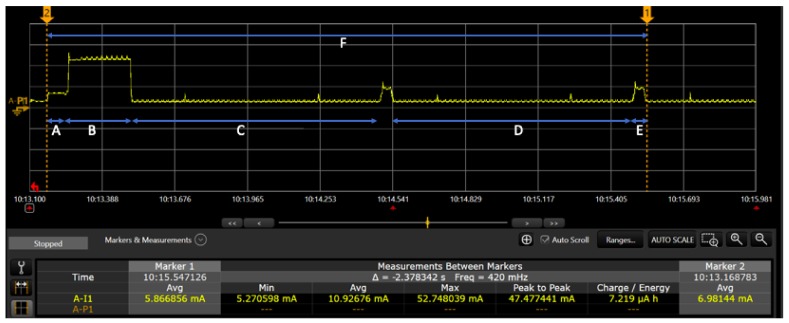
Power measurement of the LoRaWAN communication.

In the current setup we have used the battery of 15,000 mAh. It lasts for 12–13 weeks, with message frequency of five minutes and configuration as mentioned in Table 3. We did the power measurement to see the exact consumption using the Keysight N6705B power analyzer as shown in Figure 13. The letters marked in the Figure 13, represents different section of the message such as ‘A’: sensor reading; ‘B’: radio transmission time; ‘C’: one second time before opening first receive slot; ‘D’: next one second time before opening second receive slot; ‘E’: radio receive slot; ‘F’: one full message marked between two markers. The measurement for the power consumption is given in Table 6. Later, we will use this baseline power consumption analysis to compare the power consumption overhead for onboard computation (to minimize message frequency) Vs sending each message; also, the power analysis of integrating different ML algorithms.

### 5.3. Future Work

There is potential research required over the LoRaWAN-based deployment in greenhouse. The most important constraint in the current setup is power consumption. This requires a new design technique such as inclusion of energy harvesting schemes, multidisciplinary approach like machine learning, artificial intelligence and on chip data processing for energy saving. There needs to be a trade-off between the frequency of sending messages to the power required in onboard computing as given in Table 7. Also, we plan to integrate ML technique for improved crop monitoring and extending the lifetime of the network.

## 6. Conclusions

In this paper, we mainly looked into energy-efficient IoT strategies for precision agriculture in greenhouse. We have summarized the prospects and challenges for precision agriculture in greenhouse and discussed the implication of sensor box design scheme over sensor readings. It is crucial to deploy the sensors in the air circulated box for precise data and longer lifetime of the sensors. We highlight the divergent control systems used in the greenhouse and propose the sequential steps for WSN deployment and monitoring of the environment. This paper demonstrates an end-to-end complete WSN architecture and deployment of LoRaWAN-based network for the monitoring of tomato crop in the greenhouse. Progressive WSN deployment, in different phases, helps in understanding the challenges of the greenhouses and applying the mitigation scheme at every next phase. The system monitors different values like light, temperature, CO_2_ and humidity and shows the variation throughout the crop season. Therefore, it is important to keep monitoring the environmental data for precision agriculture. For future work, we would work towards the increased energy efficiency of the LoRaWAN network by energy harvesting.

## Figures and Tables

**Table 1:** Challenges for PA in greenhouse.

Greenhouse Entanglement	Salient Consequence in PA
Crop rotation	Crop-specific design management and adaptation
Movement, obstacles and building structures	Interference, deployment, routing and failure diagnosis
Cognitive mitigation techniques	Impact assessment, event-based dynamic model adaptation
Harsh environmental factor	Temperature, gas emission; variance due to shading
Plant growth and regular crop activities	Noise and sensitivity variation in greenhouse
Electronic circuit and designing	Hold out against spray, water etc., power efficient design
Sensor sensitivity and connectivity uncertainty	Impacts assessment parameter and required service quality

**Table 2:** Leveraging divergent sensors and control systems for PA in greenhouse.

Heterogeneous Sensors and Control Systems	Facilitation
Environmental control system	Heating, cooling, ventilation and crop monitoring
Illuminance sensors	Light monitoring for plant growth
Tag-based sensors	Tracking and remote identification
Multimedia sensors	Remote image capturing (insect and plant disease)
Climate sensors	Monitoring, models for prediction and early warnings
Ground sensors	Equipment control and knowledge mining
Radiation sensors	Reasoning and analytic
Weather stations	Controlled environment agriculture
Decision support system (DSS)	Inter-operability, grower specific semantic annotation
Access control sensors	Unauthorized entry into the facility

**Table 3:** LoRaWAN module configuration.

Parameter	Value
Wireless Module	CMWX1ZZABZ
Radio Chipset	SXSX1276
Microcontroller (MCU)	STM32L072CZ
Radio Frequency	868 MHz
Bandwidth	125 kHz
Coding rate	4/5
Spreading factor (SF)	9
Transmission power	14 dBm

**Table 4:** Available sensors overview.

Sensor	Type	Parameters
SHT31	Embedded on OCTA-Connect	Temperature (${}^{\circ }$C), Relative Humidity (%)
TCS34725	Embedded on OCTA-Connect	Illuminance (lux)
Cozir	Sensor shield	CO_2_ (ppm)
Water	Sensor shield with probe	Temperature (${}^{\circ }$C), Conductivity ($\mu S{\mathrm{cm}}^{-1}$), Impedance (Ω)
Strip (SHT & TCS)	Shield with expandable strip	Temperature (${}^{\circ }$C), Relative Humidity (%), Illuminance (lux)

**Table 5:** One-month (August) sensor data for tomato crop at PCH, Belgium.

Sensor	Minimum	Maximum	Average
Temperature (${}^{\circ }$C)	18.63	33.22	24.8
Relative Humidity (%)	48	91	67.5
Illuminance (lux)	274	12452	4034.17
CO_2_ (ppm)	596	1092	781
Conductivity ($\mu S{\mathrm{cm}}^{-1}$)	91	301	182

**Table 6:** Power consumption analysis for LoRaWAN message.

Phase	Time	Avg. Current (mA)
Sensor reading	82.28 ms	13.24
Radio transmission	249.8 ms	46.08
Inactive mode	5 min	6.01
One total message	2.3 s	10.92

**Table 7:** Future work.

Research Road Map	Optimization and Impact
Cognitive design technique	Electronic design and radio optimization (idle time, re-transmission etc.)
Energy consumption model	Connection strategy and power optimization techniques
Radio optimization	Passive wake up radios, time sync, data rate and coding schemes
Frequency of data transmission	Edge computing and combining multidisciplinary approach (ML, AI etc.)
Adaptive ML technique [44]	Adapt and tune future behavior and predictions.
Cost of power management scheme	Trade-off between packet transmission and computation
